# Contribution of smoking towards the association between socioeconomic position and dementia: 32-year follow-up of the Whitehall II prospective cohort study

**DOI:** 10.1016/j.lanepe.2022.100516

**Published:** 2022-09-28

**Authors:** Martina Raggi, Aline Dugravot, Linda Valeri, Marcos D. Machado-Fragua, Julien Dumurgier, Mika Kivimaki, Séverine Sabia, Archana Singh-Manoux

**Affiliations:** a Epidemiology of Ageing and Neurodegenerative diseases, Université Paris Cité, Inserm U1153, Paris, France; b Department of Biostatistics, Columbia University Mailman School of Public Health, New York, NY, USA; c Department of Epidemiology, Harvard T.H. Chan School of Public Health, Boston, MA, USA; d Cognitive Neurology Center, Lariboisière – Fernand Widal Hospital, AP-HP, Université de Paris, Paris, France; e Department of Epidemiology and Public Health, University College London, UK; f Clinicum, Faculty of Medicine, University of Helsinki, Helsinki, Finland

**Keywords:** Social inequalities, Socioeconomic position, Smoking, Mediation, Dementia

## Abstract

**Background:**

There is consistent evidence of social inequalities in dementia but the mechanisms underlying this association remain unclear. We examined the role of smoking in midlife in socioeconomic differences in dementia at older ages.

**Methods:**

Analyses were based on 9951 (67% men) participants, median age 44.3 [IQR=39.6, 50.3] years at baseline in 1985–1988, from the Whitehall II cohort study. Socioeconomic position (SEP) and smoking (smoking status (current, ex-, never-smoker), pack years of smoking, and smoking history score (combining status and pack-years)) were measured at baseline. Counterfactual mediation analysis was used to examine the contribution of smoking to the association between SEP and dementia.

**Findings:**

During a median follow-up of 31.6 (IQR 31.1, 32.6) years, 628 participants were diagnosed with dementia and 2110 died. Analyses adjusted for age, sex, ethnicity, education, and SEP showed smokers (hazard ratio [HR] 1.36 [95% CI 1.10–1.68]) but not ex-smokers (HR 0.95 [95% CI 0.79–1.14]) to have a higher risk of dementia compared to never-smokers; similar results for smoking were obtained for pack-years of smoking and smoking history score. Mediation analysis showed low SEP to be associated with higher risk of dementia (HRs between 1.97 and 2.02, depending on the measure of smoking in the model); estimate for the mediation effect was 16% for smoking status (Indirect Effect HR 1.09 [95% CI 1.03–1.15]), 7% for pack-years of smoking (Indirect Effect HR 1.03 [95% CI 1.01–1.06]) and 11% for smoking history score (Indirect Effect HR 1.06 [95% CI 1.02–1.10]).

**Interpretation:**

Our findings suggest that part of the social inequalities in dementia is mediated by smoking.

**Funding:**

NIH

**Research in context**

**Evidence before this study**

We searched PubMed for publications until February, 2022, using the search terms: “social inequalities”, “social disparities”, “socioeconomic position/status”, “ageing”, “dementia”, “Alzheimer's disease”, “mortality”, “smoking”, “cigarettes”, and “tobacco”. There is robust evidence of social inequalities in smoking and in dementia, irrespective of the measure used to assess socioeconomic position (SEP). However, whether smoking is itself associated with higher risk of dementia remains debated and few studies have examined whether smoking mediates the association between SEP and dementia. The studies that exist have examined smoking as part of lifestyle factors. Further limitations in published studies include use of conventional methods for mediation analysis precluding causal interpretation, and measurement of smoking at older ages where selection bias is more likely.

**Added value of this study**

Our analyses show a robust association between smoking in midlife and dementia over a median follow-up of 32 years. These results are important because smoking is potentially modifiable via public health policies, as demonstrated in secular decline in smoking in high-income countries. Using mediation analyses in a counterfactual framework we found socioeconomic differences in dementia and part of this association to be due to smoking (16% for smoking measured as current-, ex-, and never-smokers). Taken together, these findings 1) add to current knowledge on the role of smoking for dementia where results have been inconsistent due to late-life measures of smoking, and tobacco industry support in some studies; 2) identify smoking as a potential target for reducing social inequalities in dementia.

**Implications of all the available evidence**

Guidelines for dementia prevention typically list a number of risk factors for dementia, without attempting to establish a hierarchy among them. Socioeconomic factors are themselves associated with a number of these risk factors and the counterfactual approach for mediation used in our study might be useful to both identify risk factors for dementia and targets for reducing socioeconomic inequalities in dementia. Results of the present study suggest that social differences in smoking in midlife contribute to social inequalities in dementia at older ages.

Alt-text: Unlabelled box

## Introduction

Dementia is increasingly a public-health concern as 50 million people worldwide currently live with dementia, and this number is predicted to triple by 2050.^1^ A consistent observation in population studies is higher dementia rates in socially disadvantaged groups.^2^, ^3^, ^4^, ^5^ This is the case when social disadvantage is measured using education,^2^ occupation,^3^ or wealth.^4^ Socioeconomic factors, education in particular, feature in guidelines for dementia prevention,^6^^,^^7^ that also include a wide array of social, behavioral, and biological factors, including smoking. All risk factors in these guidelines are listed without an attempt to establish a hierarchy among them. Socioeconomic disadvantage shapes exposure to a range of risk factors,^8^^,^^9^ and this framework is potentially useful to understand socioeconomic differences in dementia.^10^, ^11^, ^12^, ^13^, ^14^

Despite secular decline in prevalence of smoking, there is robust evidence of persistence in socioeconomic differences in smoking,^15^^,^^16^ such that smoking is an important mediator of the association between socioeconomic factors and health outcomes such as mortality.^17^, ^18^, ^19^ The extent to which smoking increases the risk of dementia remains debated;^20^, ^21^, ^22^, ^23^, ^24^ with some^20^, ^21^, ^22^ but not all studies showing higher risk in smokers than non-smokers.^20^^,^^23^^,^^24^ The inconsistency in results could be due to premature death in smokers.^25^ It is also possible that combining ex-smokers and smokers in analyses^20^ dilutes the association with dementia due to dissipation over time of the harmful effects of smoking on cognitive function^21^^,^^22^ and risk of dementia.^22^ Dementia is characterized by a long preclinical period that involves multiple pathophysiological changes,^26^^,^^27^ suggesting that results from studies that assess risk factors in late-life are likely to be biased due to reverse causation.

The aim of the present study was to examine the contribution of smoking to socioeconomic differences in dementia using longitudinal data spanning three decades. We used mediation analysis for survival data within a counterfactual-based perspective^28^ to estimate the direct effect of socioeconomic position on dementia and the indirect effect mediated by smoking in midlife. In order to test the validity of our analytical approach we used this approach to examine the role of smoking in midlife in the association between socioeconomic position and mortality, the secondary outcome, as the importance of smoking for social inequalities in mortality is well known from previous studies.^17^, ^18^, ^19^

## Methods

The study objectives and analysis plan were developed prior to data manipulation as part of a grant funded by the NIH, https://reporter.nih.gov/search/sgsDe4AmXU2_O6up4UtW0Q/project-details/9713293

### Study population

The target population for the Whitehall II study was all men and women, aged 35–55, working in the London offices of twenty civil-service departments. Members of the target population were invited to participate by letter. The response rate, after excluding those who were ineligible, was 73% (74% among men, 71% among women). The study was established in 1985–1988 among 10,308 persons (6895 men and 3413 women, aged 35–55 years).^29^ Since baseline, follow-up clinical examinations have taken place approximately every four to five years. In addition to data collection within the study, participants are linked to electronic health records of the UK National Health Service (NHS). The NHS provides most of the health care in the country, including in- and out-patient care, and record linkage is undertaken using a unique NHS identifier held by all residents. Written informed consent from participants and research ethics approvals were renewed at each contact; the most recent approval was from the University College London Hospital Committee on the Ethics of Human Research, reference number 85/0938.

### Socioeconomic position (SEP)

SEP was measured using the British Civil Service grade of employment at baseline (1985–88), a 6-level variable ranging from high (administrative grades) to low (support grades) position. The measure is attributed to all employees by the employer, here the UK government and is a comprehensive marker of SEP as it reflects education, salary, social status, and level of responsibility at work.^29^

### Smoking

Smoking was measured at baseline using a series of questions on smoking status, age at smoking initiation, age at cessation among ex-smokers, number of cigarettes smoked per day, and ounces of tobacco smoked in hand-rolled cigarettes per week. From these data, three measures of smoking were extracted to examine whether the mediation effect was similar across these measures:

1. *Smoking status,* categorized as current, ex-, and never smoker. We also compared never smokers to ever smokers (combining ex- and current smokers), and current smokers to non-smokers (combining never and ex-smokers).
2. *Pack-years* of smoking obtained using the mean grams of tobacco consumed daily divided by 20 and multiplied by the number of years of smoking.
3. *Smoking history score,* to combine smoking status and pack-years of smoking using a weighted score, elaborated by Song et al.,^30^ calculated as: (smoking_i_ + 0.25 × PY_i_/10) × (2 / (1 + 0.25 × MaxPY/10)) where smoking_i_ was the individual smoking status (no/yes, coded 0/1), PY_i_ the individual value of pack-years and MaxPY the highest pack-years value in the study. This score ranges from 0 to 2, with a higher score indicating higher exposition to smoking. The weights in the score (1 for current smoking and 0.25 for pack-years) were chosen based on estimates in our study showing that the beta for the association between current smoking and dementia/mortality risk was around four times higher than that between a ten-year increment in pack-years of smoking and dementia/mortality risk.

### Primary outcome: dementia

*Dementia* cases were ascertained by linkage to three national registers (HES, the Mental Health Services Data Set, and the mortality register) up to the 31^st^ of March 2019. All-cause dementia was identified based on ICD-10 codes F00-F03, F05.1, G30, and G31. The sensitivity and specificity of dementia using the national Hospital Episodes Statistics (HES) data are 78.0% and 92.0%, respectively.^31^ The sensitivity in our study is likely to be further improved due to additional use of Mental Health Services Data Set, a national database that contains information on dementia for persons in contact with mental health services in hospitals, outpatient clinics, and the community.^32^ The NHS national mortality register was used to assess causes of death, from which dementia cases were identified. Date of dementia was set at the first record of dementia diagnosis using all three databases.

### Secondary outcome: mortality

Death from any cause was the secondary outcome. Mortality data until 31st March 2019 were drawn from the NHS national mortality register using the participant's unique NHS identification number.

### Covariates

*Sociodemographic factors* included age (continuous variable), sex (male/female), ethnicity (Caucasian, other), and education (partial secondary school or lower, secondary school, university and higher degree); the latter to account for early life socioeconomic circumstances and differences in educational achievement.

### Statistical analysis

In preliminary analyses we examined the shape of the association of SEP in six categories with smoking status (current, ex-, and never smoker), dementia, and mortality (incidence rate per 1000 person-years) in order to ascertain whether SEP could be used as a continuous variable, ranging from 0 (highest SEP) to 1 (lowest SEP), using increments of 1/6 so that a change of one point (change from 0 to 1) reflected risk in the lowest compared to highest SEP group. Comparison of goodness of fit of the categorical and continuous SEP measure using AIC and BIC criteria for associations of SEP with smoking (logistic regression for smoking status and linear regression for pack-years of smoking and smoking history score), incident dementia and mortality (Poisson regression) suggested better fit with the continuous SEP measure.

**Figure 1:**
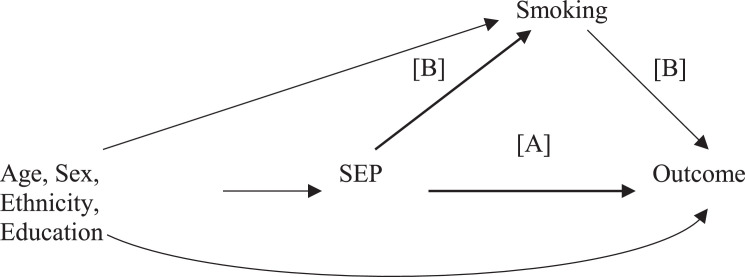
**Directed Acyclic Graph (DAG) for the analysis of the contribution of smoking to the association between socioeconomic position (SEP) and dementia/mortality**. [A] Reflects the “direct” effect of SEP on the outcome (dementia and mortality, analyzed in separate models). This is the effect not mediated by smoking. [B] Reflects the indirect effect of SEP on the outcome, effect that is mediated by smoking.

Standard approaches to mediation, the difference-of-coefficients or product-of-coefficients method, provide accurate estimates for linear models without interactions,^28^^,^^33^ motivating our use of a counterfactual framework for causal mediation. The hypothesized causal structure of the association between SEP (the exposure) and the outcome (dementia and then mortality), with smoking as a mediator is shown as a Directed Acyclic Graph in Figure 1, and counterfactual effect definitions in eTable 1. The mediating role of smoking within the counterfactual framework was examined in a single analysis with survival data under a regression-based approach.^28^ The counterfactual framework involves an explicit causal structure and allows simultaneous estimation of direct (the SEP and outcome association not due to the mediator), indirect (association due to the mediator), and total effects (direct and indirect effects).

The exposure (SEP), confounders (age, sex, ethnicity, and education), and the mediator (smoking measures) were measured at baseline, allowing us to assume that confounders of the mediator-outcome association were unaffected by the exposure. Interaction terms between SEP and smoking (*p*>0.05) did not suggest the association between smoking and dementia to differ by SEP. This led us to estimate the natural direct and indirect effects where the natural direct effect can be interpreted as the effect of SEP on the outcome (change in outcome when exposure moves from 0 (highest SEP) to 1 (lowest SEP) with the mediator (smoking) being distributed as in the reference group, here high SEP. The natural indirect effect describes mediation, that is the part of the association between SEP and dementia due to smoking. The counterfactual effects were estimated as a combination (sums and products) of the regression coefficients obtained from:

i) A multinomial logistic model (smoking status) or a linear model (pack-years and smoking history score) for the association between the exposure (SEP, continuous scale) and the mediator (smoking measure), adjusted for age, sex, ethnicity, and education. Pack-years of smoking and smoking history score were square root transformed to normalize their skewed distributions.
ii) A Cox proportional hazard model for the outcome (dementia or death), including the exposure (SEP, continuous scale), the mediator (separate models for each measure of smoking), and confounders (age at baseline, sex, ethnicity, and education). For dementia analysis, participants were censored at the date of dementia diagnosis, death, or 31^st^ March 2019, and for mortality the date of death or 31^st^ of March 2019. The proportional hazards assumption was verified by plotting Schoenfeld residuals.

To compute counterfactual effect estimates and 95% Confidence Interval (CI), obtained via delta method, we used the R command ‘cmest’, included in the R package CMAverse.^34^ All the counterfactual effects were reported on the hazard ratio scale conditional on covariates. The proportion of association mediated by smoking was calculated as Direct Effect × (Indirect Effect - 1)/(Total Effect - 1).

Additional analyses (see eMethods 1 for further description) to examine robustness of findings: One, we used Accelerated Failure Time (AFT) model^28^ to reanalyse the mortality outcome as Cox proportional hazards mediation analysis requires the outcome to be a rare event and there were too many deaths for this assumption to hold. Two, we used the simulation extrapolation (SIMEX) approach to correct for possible measurement error in the mediator.^35^ This method consists of adding increasing levels of measurement error in smoking to the simulations to observe the trend in the parameter estimates. Three, inverse probability of censoring weighting (IPCW) approach was used to address selection bias resulting from death occurring before the possibility of dementia diagnosis.^36^ All the analyses were performed with R software (version 4.0.3).

### Role of the funding source

The funders of the study had no role in study design, data collection, data analysis, data interpretation, or writing of the report. MR and AD had full access to all the data and had final responsibility to submit for publication.

## Results

The analyses were based on 9951 (96.5%) of the 10,308 participants recruited to the study in 1985–1988 (flow chart in eFigure 1). Table 1 summarizes participants’ characteristics at baseline by dementia and death status by the end of follow-up (31^st^ of March 2019). Of 9951 participants, 628 were diagnosed with dementia (of whom 320 were first diagnosed in HES, 294 in the Mental Health Services Data Set, and 14 in the mortality register). A total of 2110 participants died, the median follow up in those alive at the end of the follow-up was 32·0 [IQR=31.5, 32.8] and among those who died it was 24.0 [IQR=16.9, 28.4] years. Mean age at dementia diagnosis was 76.8 (SD 6.0) years and at death 70.5 (SD 9.8) years. In eTable 2 participants’ baseline characteristics are summarized according to smoking status; current smokers were more likely to be female, have lower education and SEP. The distribution of smoking (eFigure 2) and incidence rates per 1000 person years of dementia and mortality (eTable 3) by SEP in six categories suggest fairly linear associations.

**Table 1:** Characteristics of participants at baseline (1985–1988) according to dementia and death status at the end of the follow-up (31^st^ of March 2019).

	Total sample	Dementia status			Death status		
Characteristics		No dementia	Dementia	*p*-value	Alive	Death	*p*-value
	*N*=9951	*N*=9323	*N*=628		*N*=7841	*N*=2110	
Age (years), Mean (SD)	44.9 (6.0)	44.6 (6.0)	49.9 (4.8)	<0.0001	44.0 (5.8)	48.5 (5.7)	<0.0001
Male sex	6669 (67.0)	6307 (67.6)	362 (57.6)		5293 (67.5)	1376 (65.2)	0.05
White ethnicity	8960 (90.0)	8421 (90.3)	539 (85.8)		7072 (90.2)	1888 (89.5)	0.35
Education				<0.0001			<0.0001
Partial secondary school or lower	4712 (47.4)	4336 (46.5)	376 (59.9)		3547 (45.2)	1165 (55.2)	
Secondary school	2650 (26.6)	2517 (27.0)	133 (21.2)		2109 (26.9)	541 (25.6)	
University and higher degree	2589 (26.0)	2470 (26.5)	119 (18.9)		2185 (27.9)	404 (19.1)	
SEP category^a^				<0.0001			<0.0001
1 (high)	1099 (11.0)	1038 (11.1)	61 (9.7)		846 (10.8)	253 (12.0)	
2	1831 (18.4)	1732 (18.6)	99 (15.8)		1519 (19.4)	312 (14.8)	
3	1384 (13.9)	1318 (14.1)	66 (10.5)		1139 (14.5)	245 (11.6)	
4	1927 (19.4)	1838 (19.7)	89 (14.2)		1600 (20.4)	327 (15.5)	
5	1489 (15.0)	1402 (15.0)	87 (13.9)		1128 (14.4)	361 (17.1)	
6 (low)	2221 (22.3)	1995 (21.4)	226 (36.0)		1609 (20.5)	612 (29.0)	
Smoking status				0.31			<0.0001
Never smoker	5034 (50.6)	4719 (50.6)	315 (50.2)		4180 (53.3)	854 (40.5)	
Ex-smoker	3133 (31.5)	2946 (31.6)	187 (29.8)		2487 (31.7)	646 (30.6)	
Current smoker	1784 (17.9)	1658 (17.8)	126 (20.1)		1174 (15.0)	610 (28.9)	
Pack-years of smoking,^b^ Mean (SD)	16.6 (14.4)	16.5 (14.5)	20.1(17.1)	<0.0001	14.8 (13.1)	22.2 (17.5)	<0.0001
Smoking history score,^b^ Mean (SD)	0.6 (0.5)	0.6 (0.5)	0.7 (0.5)	0.002	0.5 (0.5)	0.8 (0.5)	<0.0001

Values are N (%) unless otherwise indicated.

a SEP categories: category 1 = unified grades 1-6 of the civil service, 2 = unified grade 7, 3 = senior executive officer, 4 = higher executive officer, 5 = executive officer, 6 = clerical officer/office support.

b In ex- and current smokers (*N*=4917). Abbreviations: SD, standard deviation; SEP, socioeconomic position (measured using occupational position).

In analyses adjusted for age, sex, ethnicity, and education, the odds ratio of being a current smoker in the low compared to the high SEP group was 4.60 [95% CI 3.64–5.83]; a similar pattern was observed for pack-years of smoking and smoking history score (Table 2). The associations of measures of smoking with dementia in analyses adjusted for confounders (age, sex, ethnicity, and education) are presented in Table 3 (top panel). Compared to never smokers, smokers (Hazard Ratio, HR 1.44 [95% CI 1.17–1.77]) but not ex-smokers (HR 0.96 [95% CI 0.80–1.15]) had a higher risk of dementia. The lack of higher risk in ex-smokers was confirmed in reanalysis of smoking status by combining ex- and current smokers and then never and ex-smokers. Every additional unit increment in square root transformed pack-years of smoking (HR 1.05 [95% CI 1.02–1.09]) and smoking history score (HR 1.39 [95% CI 1.16–1.68]) were also associated with higher risk of dementia.

**Table 2:** Association between SEP and measures of smoking.

	Multinomial OR for ex- vs never smoker (95% CI)	*p*-value	Multinomial OR for current vs never smoker (95% CI)	*p*-value
Lowest vs Highest SEP	1.04 (0.87–1.25)	0.66	4.60 (3.64–5.83)	<0.0001
				

	β (95% CI) for pack-years of smoking^a^	*p*-value	β (95% CI) for smoking history score^b^	*p*-value
Lowest vs Highest SEP	0.81 (0.64 to 0.98)	<0.0001	0.20 (0.17 to 0.24)	<0.0001
				

a Square root of pack-years of smoking.

b Square root of smoking history score. Abbreviations: OR, odds ratio; CI, confidence interval; β beta coefficient, SEP, socioeconomic position (measured using occupational position).

**Table 3:** Associations of measures of smoking with dementia (N dementia/total = 628/9951) and mortality (N deaths/total = 2110/9951).

Health outcomes	Measures of smoking	Unadjusted for SEP		Adjusted for SEP	
		HR (95% CI)^a^	*p*-value	HR (95% CI)^a^	*p*-value
Dementia					
	Smoking status				
	Never smoker	Ref		Ref	
	Ex-smoker	0.96 (0.80–1.15)	0.63	0.95 (0.79–1.14)	0.57
	Current smoker	1.44 (1.17–1.77)	0.001	1.36 (1.10–1.68)	0.005
					
	Never smoker	Ref		Ref	
	Ex- and current smoker	1.11 (0.95–1.30)	0.21	1.08 (0.92–1.27)	0.34
					
	Never and ex-smoker	Ref		Ref	
	Current smoker	1.46 (1.20–1.78)	<0.0001	1.39 (1.14–1.69)	0.001
					
	Pack-years of smoking^b^	1.05 (1.02–1.09)	0.004	1.04 (1.01–1.08)	0.02
					
	Smoking history score^c^	1.39 (1.16–1.68)	<0.0001	1.33 (1.10–1.60)	0.003
					
Mortality					
	Smoking status				
	Never smoker	Ref		Ref	
	Ex-smoker	1.15 (1.04–1.28)	0.007	1.15 (1.04–1.28)	0.01
	Current smoker	2.33 (2.09–2.58)	<0.0001	2.23 (2.00–2.48)	<0.0001
					
	Never smoker	Ref		Ref	
	Ex- and current smoker	1.53 (1.40–1.68)	<0.0001	1.50 (1.37–1.64)	<0.0001
					
	Never and ex-smoker	Ref		Ref	
	Current smoker	2.19 (1.99–2.41)	<0.0001	2.10 (1.91–2.34)	<0.0001
					
	Pack-years of smoking^b^	1.14 (1.12–1.16)	<0.0001	1.13 (1.11–1.15)	<0.0001
					
	Smoking history score^c^	2.22 (2.02–2.44)	<0.0001	2.14 (1.95–2.36)	<0.0001
					

a Analyses using Cox regression with time as timescale, adjusted for age at baseline, sex, ethnicity and education.

b Square root of pack-years modelled as a continuous variable.

c Square root of smoking history score modelled as a continuous variable. Abbreviations: HR, hazard ratio; CI, confidence interval; SEP, socioeconomic position (measured using occupational position).

The association between measures of smoking and mortality (Table 3, bottom panel) adjusted for confounders were somewhat stronger and compared to never smokers the risk of mortality was higher in both ex- (HR 1.15 [95% CI 1.04–1.28]) and current-smokers (HR 2.33 [95% CI 2.09–2.58]). Combining ex-and current smokers showed them to have higher risk of mortality (HR 1.53 [95% CI 1.40–1.68]) compared to never smokers. Findings were similar using the AFT model for mortality (eTable 4). For example, current smokers had 31% (100×(1–0.69); mean survival ratio 0.69 [95% CI 0.66–0.73]) lower survival time compared with never smokers. For both dementia and mortality, further adjustment for SEP did not substantially modify the findings.

The results of the mediation analyses for the role of smoking in the association of SEP with dementia (top panel) and mortality (bottom panel) while accounting for confounders are presented in Table 4. The HR reflecting the direct effect of SEP on dementia (not mediated by smoking) was 1.86 [95% CI 1.35–2.57], 1.90 [95% CI 1.38–2.62], and 1.86 [95% CI 1.35–2.57], respectively for smoking status, pack-years of smoking, and smoking history score as the mediator. The HR reflecting the indirect effect for the measure of smoking was 1.09 [95% CI 1.03–1.15], reflecting 16% of the total effect of SEP (HR 2.02 [95% CI 1.47–2.78]). When considering pack-years of smoking the proportion mediated was 7% (indirect effect HR 1.03 [95% CI 1.01– 1.06]), while for smoking history score as mediator the indirect effect HR was 1.06 [1.02–1.10], with the proportion mediated being 11%.

**Table 4:** Decomposition of the association between SEP and health outcomes to examine the role of smoking.

Health outcomes	Mediation with	Hazard ratio (95% CI)^a^	*p*-value	% mediation^b^
Dementia	Smoking status (3 categories)			
	Total Effect	2.02 (1.47–2.78)	<0.0001	
	Direct Effect	1.86 (1.35–2.57)	<0.0001	
	Indirect Effect	1.09 (1.03–1.15)	0.004	16%
	Pack-years of smoking^c^			
	Total Effect	1.97 (1.43–2.71)	<0.0001	
	Direct Effect	1.90 (1.38–2.62)	<0.0001	
	Indirect Effect	1.03 (1.01–1.06)	0.02	7%
	Smoking history score^d^			
	Total Effect	1.97 (1.43–2.71)	<0.0001	
	Direct Effect	1.86 (1.35–2.57)	<0.0001	
	Indirect Effect	1.06 (1.02–1.10)	0.004	11%
Mortality	Smoking status (3 categories)			
	Total Effect	1.95 (1.64–2.32)	<0.0001	
	Direct Effect	1.60 (1.34– 1.90)	<0.0001	
	Indirect Effect	1.22 (1.17–1.27)	<0.0001	37%
	Pack-years of smoking^c^			
	Total Effect	1.86 (1.57–2.22)	<0.0001	
	Direct Effect	1.68 (1.42–2.00)	<0.0001	
	Indirect Effect	1.11 (1.08–1.14)	<0.0001	21%
	Smoking history score^d^			
	Total Effect	1.86 (1.57– 2.21)	<0.0001	
	Direct Effect	1.59 (1.34–1.90)	<0.0001	
	Indirect Effect	1.17 (1.13–1.21)	<0.0001	31%

a The HR reflects gradient in dementia across the SEP scale, estimates are conditional on mean age at baseline, male sex, white race and education at partial secondary school or lower; 95% CI calculated using the delta method. Differences in Total effects of SEP for each outcome result from combination of estimates of the Direct and Indirect effects.

b Calculated as *Direct Effect x (Indirect Effect - 1)/(Total Effect - 1).*

c Square root of pack-years modelled as a continuous variable.

d Square root of smoking history score modelled as a continuous variable. Abbreviations: HR, hazard ratio; CI, confidence interval; SEP, socioeconomic position (measured using occupational position).

The total effect of the association between SEP and mortality (HR between 1.86 and 1.95) was similar to that between SEP and dementia (HR between 1.97 and 2.02) but a larger proportion of the association between SEP and mortality was explained by smoking: 37%, 21%, and 31% for smoking status, pack-years of smoking, and smoking history score respectively. This led to a stronger indirect effect. For example, the HR for the indirect effect using smoking status as the mediator was 1.22 [95% CI 1.17–1.27]. Findings were similar using the AFT model for the association between SEP and mortality (eTable 5).

Further analyses accounting for measurement error in the smoking history score suggested that increasing measurement error yielded stronger mediation for dementia, ranging from 11% in the observed data to 17% when measurement error was highest in the simulations (eTable 6); a similar pattern was also observed for mortality (eTable 7). Analyses aimed at taking selection due to death in the analyses of dementia as an outcome (eTable 8) did not substantially change the main results.

## Discussion

There are three key findings from this longitudinal study of nearly 10,000 participants with data on three types of smoking measures and a long follow-up for dementia and mortality. One, smoking in midlife was associated with an increased risk of dementia at older ages, irrespective of the manner in which smoking was measured. Two, the social gradient in dementia was partially mediated by smoking (proportion mediated: 16% for smoking status, 7% for pack-years of smoking, and 11% for smoking history score). Three, smoking explains more of the association of SEP with mortality than that with dementia as seen in the indirect effects of SEP on dementia being smaller than those for mortality.

There is increasing evidence of a higher risk of dementia among smokers.^20^, ^21^, ^22^ Meta-analyses show current smokers to have an increased risks of dementia compared with never smokers while there was no robust evidence of an increased risk among ex-smokers.^20^ Combining smokers and ex-smokers and comparing them to never smokers yields null^20^ associations with dementia. Studies also show that stopping smoking in midlife is associated with slower cognitive decline^21^ and lower risk of dementia compared to smokers.^22^ Our results reflect these findings, with no excess risk of dementia among ex-smokers although there remained a higher risk of mortality in this group.

There are at least two sources of bias in studies reporting no association between smoking and dementia. One, tobacco-industry affiliation appears to affect results. Pooled estimates from 14 cohort studies without an affiliation with the tobacco industry found higher risk of dementia in smokers whereas three cohort studies affiliated with the tobacco industry found smoking to have a protective association with dementia.^23^ The second source of bias is the assessment of smoking at older ages, typically in studies with a short follow-up or in case-control studies. Meta-analysis of case control studies shows no association between smoking and dementia.^20^^,^^23^ A recent study based on data from low and middle income countries on adults 65 years and older at baseline and followed up for 3·8 years showed no significant association between smoking and dementia.^24^ Survival bias^25^ may lead a selected group of smokers to be included in studies that measure smoking late in life.

The role of smoking in explaining the social gradient in dementia has been examined in a few studies using mediation analysis, either with a focus on smoking alone^10^ or including smoking as part of health and lifestyle factors.^11^, ^12^, ^13^, ^14^ All the studies on the mediating role of lifestyle factors were based on adults 65 and older at baseline and they reported 17–52% of the association between socioeconomic factors and dementia was explained by lifestyle factors. Two of these studies^13^^,^^14^ examined the role of smoking itself and did not find any strong evidence of indirect effects. One study specifically focused on smoking found it to explain 27% of the association between socioeconomic factors and brain volume.^10^ Although this study was based on middle-aged adults (mean age ∼55 years), the cross-sectional nature of the study remains an important limitation. A further limitation is the sub-optimal methodology adopted to estimate the indirect effects. With the exception of one study,^13^ which like in our study adopted the counterfactual framework to decompose the total effect, all other studies used conventional approaches such as the difference-of-coefficients and product-of-coefficients methods.

The contribution of smoking to socioeconomic inequalities in mortality has been widely investigated.^17^, ^18^, ^19^ A previous study based on data from the Whitehall II study found smoking to explain 32% of the association between SEP and mortality in analyses using the conventional framework.^17^ A longitudinal study based on US adults followed for ten years estimated that less than 25% of the total association between socioeconomic status and all-cause mortality was explained by smoking.^18^ Findings were similar in a study based on 14 European countries which showed the proportion attributable to smoking in socioeconomic differences in mortality ranged from 19% to 55% among men and from −1% to 56% among women.^19^

In the present study, while smoking was associated with an increased risk of dementia, its contribution to explaining social inequalities in dementia was modest although in additional analyses we show it to be underestimated due to possible measurement error in the smoking variable. In our analyses smoking status had stronger indirect effects than smoking history score or pack-years of smoking, possibly because current smoking status is likely to reflect long-term previous and future behavior, leading to stronger associations with both SEP and dementia. The association between smoking and dementia could be explained by the fact that smoking is an important risk factor for cardiometabolic and other chronic diseases^37^ which in turn are related to a higher risk of dementia.^38^ The increased risk dementia among smokers may involve other pathways. Smoking negatively affects cortical thickness,^7^ and intracranial brain volume,^10^ reflecting accelerated cognitive aging. The modest indirect effects found in our study may be due to the fact that dementia is a multi-factorial disease^38^ and the association between socioeconomic factors and dementia is likely to be mediated by a range of other socioeconomically patterned dementia risk factors (e.g. hypertension, depression, obesity, and cardiovascular events)^11^, ^12^, ^13^, ^14^ or other biological mechanisms.

To our knowledge, this study is the first longitudinal study to examine whether smoking in midlife explains socioeconomic disparities in dementia. Using data from the Cognitive Function and Ageing Study, a recent report found no evidence that diagnosis among people with dementia differed across groups classified by educational attainment or by area-level deprivation.^5^ Thus, access to care is not likely to be explanation for social inequalities in dementia. The primary strengths of this study include a long follow-up and the method used to estimate mediation. The counterfactual approach overcomes several limitations of the traditional approach to mediation analysis, such as the ability to handle nonlinear models, simple decomposition of direct and indirect effects, and assessment of an interaction between the exposure and the mediator. Use of the causal mediation framework allowed us to show that the association of smoking with dementia is similar in all SEP groups. Despite the term “causal” it is best to see the analyses reported here as an attempt to decompose the total effect into natural direct and natural indirect effects, fundamental to understanding how socioeconomic factors affect the risk of dementia. As the precise risk factors for dementia remain unclear we did not attempt to include other possible mediators which are likely to be important as smoking mediated only a modest proportion of the SEP-dementia association. It is also possible that our analysis does not consider all possible confounders of the exposure–outcome, exposure–mediator, mediator–outcome, and unmeasured mediator–outcome confounders affected by the exposure. Viewed from a descriptive perspective, our findings highlight the hierarchy in the risk factors for dementia, in that social patterning of risk factors is important to consider in research on risk factors for dementia.

As most longitudinal cohort studies, this study has several limitations. These include smoking being self-reported, potential presence of unmeasured confounding, especially for the association between the mediator and the outcome. Another limitation is the study participants being healthier than the general population, both in terms of risk factor profiles and incidence of disease. This is reflected in lower incidence of dementia; in our study this is also due to the participants being only 66 to 89 years old at the end of the follow-up. It is worth noting that analyses based on data from longitudinal cohort studies do not necessarily bias risk factor-disease associations.^39^ For example, the association between cardiovascular risk factors and incidence of cardiovascular disease in the Whitehall II study is similar to that in general population studies.^40^ A further limitation is the use of electronic health records to ascertain dementia and mortality. This method is not “gold-standard” and is likely to miss milder cases of dementia,^31^ and possibly cases with impairment in domains other than memory and orientation,^5^ but it has the advantage of dementia status being available on all participants in the study rather than only those who continue to participate in face to face screening over the follow-up although data on causes of dementia are incomplete and did not allow analyses on dementia sub-types. A final limitation is that all smoking measures were drawn from baseline and changes in smoking over the follow-up was not considered. This might bias findings but the direction of the bias is unclear. There are no straightforward solutions as the preclinical phase of dementia is 15 to 20 years and ideally risk factors ought to be measured before the onset of disease, leading to our use of an early measure of smoking.^41^

Dementia has a tremendous impact on the person, their family, and health and social care systems highlighting the importance of identifying and intervening on modifiable risk factors for dementia. Our findings show smoking to be a risk factor for dementia. While smoking in high-income countries has declined considerably it remains a major risk factor in low and middle-income countries where dementia incidence is rising rapidly. Ageing of populations in these countries makes smoking an important prevention target. There is robust evidence of social inequalities in dementia, even in high income countries. Using a counterfactual approach to determine “causal” effects we found smoking to explain part of the socioeconomic differences in dementia. These findings suggest that reducing the social gradient in smoking is likely to contribute to reducing socioeconomic inequalities in dementia.

## Contributors

Conceptualization: MR, AD, SS, ASM.

Methodology: MR, AD, LV, SS, ASM.

Investigation: MR, AD, LV, MDM-F, JD, MK, SS, ASM.

Validation: AD, SS, ASM.

Formal analysis: MR, AD.

Access and verified the data: MR, AD.

Data Curation: AD.

Writing –original draft preparation: MR, ASM.

Writing –review and editing: MR, AD, LV, MDM-F, JD, MK, SS, ASM.

Visualization: MR, AD, SS, ASM.

Supervision: AD, SS, ASM.

Funding acquisition: MK, ASM.

## Data sharing statement

Whitehall II data cannot be shared publicly because of constraints dictated by the study's ethics approval and IRB restrictions. The Whitehall II data are available for sharing within the scientific community. Researchers can apply for data access at https://www.ucl.ac.uk/epidemiology-health-care/research/epidemiology-and-public-health/research/whitehall-ii/data-sharing.

## Declaration of interests

We declare no competing interests.

## Appendix Supplementary materials

Image, application 1
