# Supplementary material for: Contribution of smoking towards the association between socioeconomic position and dementia: 32-year follow-up of the Whitehall II prospective cohort study
Source: Lancet Reg Health Eur. 2022 Sep 28;23:100516. doi: 10.1016/j.lanepe.2022.100516 (PMC9523395; doi:10.1016/j.lanepe.2022.100516)
Supplement: Supplementary file 1 [file mmc1.docx]

**eFigure 1. Flow chart**

**eFigure 2. Distribution of smoking across socioeconomic position (SEP) categories**

**eMethods 1. Description of statistical models and methods for the additional analyses**

**eTable 1. Definitions of direct, indirect, and total effects in the counterfactual framework**

**eTable 2. Characteristics of participants at baseline (1985–1988) according to smoking status**

**eTable 3. Incidence Rate (IR) of dementia and mortality across socioeconomic position (SEP) categories**

**eTable 4. Association between measures of smoking and mortality using the Accelerated Failure Time (AFT) model (N deaths/total = 2110/9951)**

**eTable 5.** **Decomposition of the association between SEP and mortality to examine the role of smoking** **using the** **Accelerated Failure Time (AFT) model (N deaths/total = 2110/9951)**

**eTable 6. The role of smoking in the association between SEP and dementia, allowing for measurement error^a^ in the smoking history score^b^**

**eTable 7. The role of smoking in the association between SEP and mortality, allowing for measurement error**^a^ **in the smoking history score^b^**

**eTable 8. Decomposition of the association between SEP and dementia to examine the role of smoking using inverse probability weighting^a^**
